# Validity and reliability evidence for the scale of distance education satisfaction of medical students based on item response theory (IRT)

**DOI:** 10.1186/s12909-022-03153-9

**Published:** 2022-02-11

**Authors:** Çetin Toraman, Engin Karadağ, Murat Polat

**Affiliations:** 1 Faculty of Medicine, Department of Medical Education, Çanakkale Onsekiz Mart University, Çanakkale, Turkey; 2 Present Address: Faculty of Education, Department of Education Management, Akdeniz University, Antalya, Turkey; 3 Present Address: Foreign Languages Department, Anadolu University, Eskisehir, Turkey

**Keywords:** Scale Development, Distance Education Satisfaction, Item Response Theory

## Abstract

**Background:**

The objective of this study is to reveal on the basis of the item response theory (IRT) the validity and reliability evidence for the data obtained from the scale prepared to determine the satisfaction with distance education in students studying in medical schools.

**Methods:**

This is a quantitative study exploring IRT and measurement invariance evidence in developing a scale. The scale whose IRT evidence was explored was the Distance Education Satisfaction Scale (DESS). The data were obtained from 1332 medical school students who were studying at various universities. The data were analysed using the confirmatory factor analysis (CFA), multidimensional and unidimensional IRT, and measurement invariance.

**Results:**

A 20-item construct with 3 sub-factors was found for DESS. This construct was unable to pass the iteration limit in the multidimensional IRT analysis. A unidimensional IRT was used assuming that the 3 sub-factors were locally independent.

**Conclusions:**

The least informative items were item 23, 24 and 25 in Factor 1, item 3 in Factor 2, and items 13 and 18 in Factor 3. The most informative items of DESS were those that had adaptive, useful expressions that had meaningful content and were able to provide educator support, which are the properties emphasized in the literature with respect to satisfaction with distance education. A measurement invariance test made based on gender revealed that DESS satisfied measurement invariance by meeting the compliance indexes required for configural, metric, scalar and strict invariance as recommended in the literature. The results showed that it is possible to make comparisons on the basis of gender using DESS.

**Supplementary Information:**

The online version contains supplementary material available at 10.1186/s12909-022-03153-9.

## Background

In the end of December 31, 2019, China had reported a new type of pneumonia infected by coronavirus (COVID-19) in Wuhan to the World Health Organization (WHO) and this caused serious diseases and deaths [1]. In January 2020, the COVID-19 infection was already a pandemic within a few weeks affecting more than 160 countries, leaving the whole world facing a global problem which had never been faced in recent history. Since the virus was spreading very fast and was so dangerous that it caused deaths in certain age groups and/or people who had some prior diseases, the whole world rapidly took serious measures from closing down non-critical workplaces to suspending education in the periods following this fast spread of the virus. In this process, the primary, secondary and high schools as well as universities were shut down temporarily and a decision was made to continue education using distance education means in many other countries.

The first COVID-19 case was reported on March 11, 2020 in Turkey, which is currently among the top 10 countries in terms of the number of COVID cases. Next, a number of measures were taken in the days following this date by various institutions and establishments. First, education was suspended as of March 16, 2020 for a period of three weeks in all schools including preschool education, secondary education and higher education institutions. The Council of Higher Education (CHE) [2] also decided on the suspension of education in this period of three weeks covering all associate degree and undergraduate students attending formal education programs as well as associate degree and undergraduate students having apprenticeship, internship and practical trainings in health, education, applied sciences and engineering programs. When the number of cases increased rapidly in Turkey and it was realized that the pandemic would last long, CHE made a decision on March 26, 2020 to continue the learning-teaching process only using distance education, open education and digital teaching facilities.

The rapid spread of COVID-19 pandemic coerced all educators across the world to continue their classes over various platforms that are the gifts of modern and advanced technology to the educators. This decision required higher education councils, faculty members and students to adapt the situation as soon as possible [3]. The General Directorate of UNESCO said that ‘a territory without a map has been entered, meaning the borderlines were crossed’ implying the distance education platforms. It was stressed that all countries should work together to find solutions involving the settings with high technology, low technology and no technology to ensure continuity of learning in this process [3]. Tamrat and Teferra [4] argue that very little research has been conducted on distance online learning systems (either hybrid or completely based on distance education) which have been used in pandemic have not been analysed satisfactorily in these days when almost entire education institutions have been closed. Therefore, it is quite important to evaluate student satisfaction which is defined as the attitude arising from evaluation of experiences, services and facilities in this sense [5] from distance education.

### Rationale

Engineering, architecture, teacher training, archaeology, sociology, philosophy, nursing, medical education and many other disciplines too many to be counted here have unique characteristics of the education given at university level. Particularly in medical education, which is the core of this research, basic sciences (for example, medical biochemistry, medical biology, anatomy, etc.) are given preclinic practice where invitro training is carried out. On the other hand, the clinic (invivo) where the knowledge and skills of medical specialties are gained, the level of competence of the acquired knowledge and skills is critical. It involves the intern doctor educational process carried out in order to achieve the medical education’s main objective, hands-on practice. Moreover, medical education is supposed to prioritize both problem-based (PBL) and community-based learning, which require horizontal and vertical integration, a holistic view as well as learning the special knowledge and skills of the intended skills [6]. Finally, each department at the higher education level has been affected by the distance education applications carried out during the pandemic period considering their own educational characteristics. Thus, distance education practices carried out within the scope of medical education have also affected the satisfaction of medical faculty students due to its characteristics.

All in all, evidence from literature reveals that learner satisfaction is depicted as a non-objective criterion including how vigorous a learning-teaching setting enhances the student success [7] and could be considered as a genuine sign of learner commitment [8]. Therefore, student satisfaction from a course might determine the quality and in-depth reflection of e-learning applications [9–11]. This fact was also emphasized in literature and satisfaction levels of online learners was studied extensively [12, 13]. In addition, it was concluded that the Medical School Students' Attitudes towards Online Learning Scale [14], E-Learning Readiness of Medical Students [15], Student Interaction and Satisfaction in a Blended Learning Environment [16] scales might be mentioned as care-taking evidences for the need to do research on distance learning. However, no research has been found to scale the satisfaction level of medical students from distance education. Findings of this research; therefore, could be useful for the administrators and faculty staff of medical faculties while planning and evaluating their educational programs.

### Classical Test Theory (CTT) & Item Response Theory (IRT)

Measurement tools can be developed according to different theories. CTT is one of them and a widely used test development theory in all over the world; thus, traditionally developed measurement tools are mostly discussed in the context of CTT. The ease and practicality of calculations in the scale development process makes CTT stand out among the others. However, measurement tools developed according to CTT have a number of limitations. For example, the psychometric properties of a tool developed according to CTT depend on the group to which the tool is applied. In addition, a single standard error value can be obtained for a whole group in measurement tools developed according to CTT. In IRT, on the other hand, item parameters are independent of the respondent group, and similarly, group characteristics are independent of the item sample. In addition, a unique standard error estimation is possible for each participant [17]. For this reason, the objective of this study is to reveal the validity and reliability evidence of the scale prepared to determine the satisfaction levels of medical school students studying in distance education on the basis of the item response theory (IRT).

## Methods

### Data collection tool

The “Distance Education Satisfaction Scale (DESS)” was used in the study [18]. As a result of the exploratory factor analysis conducted by the researchers, the construct of the scale included 37 items and 7 factors. A confirmatory factor analysis was also conducted for this 7-factor construct. The goodness of fit indices obtained from CFA (RMSEA = 0.06, GFI = 0.91, AGFI = 0.86, NFI = 0.91, CFI = 0.91) are within acceptable limits as the literature suggests limits which fit the findings of DESS [19–24]. Cronbach Alpha reliability values of the scale were between 0.79 and 0.96. The 37 items were scored from 1 to 10 in Likert type scoring. It should also be noted that the previously developed form of DESS adopted the aim of determining the satisfaction levels of all university students from distance education. In this study, DESS was examined on the basis of IRT only to determine the psychometric properties of medical school students in the context of measuring their satisfaction from distance education.

### Participants

In this study, the data on the satisfaction levels of medical school students studying in distance education were obtained from 1332 students of various universities, different grades and genders. E-mail invitations to participate in the research were sent to the medical faculty students of all universities in Turkey. Since participation in the study was voluntary, only medical school students who gave consent for voluntary participation were included in the study. In this respect, the research sample became a convenience sample. Different universities have different numbers of medical students in Turkey. That’s why, in this sample, careful attention was paid to the proportional representation of the student numbers of the universities. The data were divided into two. The first dataset included 30% (*n* = 411) of all data and the second 70% (*n* = 934). The first dataset was used for exploratory factor analysis. When determining the number of individuals to be included in this group, the minimum participant numbers recommended by the literature to be included in the sample in order to conduct an Exploratory Factor Analysis (EFA) were taken as reference [25–30]. The second group was used for Confirmatory Factor Analysis (CFA), IRT analyses and measurement invariance. Some characteristics of these groups are shown in Table 1.

**Table 1:** Demographic characteristics of the participants

Variable		EFA f (%)	CFA, IRT and Measurement Invariance f (%)
**Gender**	Female	226(55)	530(56.7)
	Male	185(45)	404(43.3)
**Years**	Preparatory Class	9(2.2)	26(2.8)
	Year 1	119(29)	250(26.8)
	Year 2	114(27.7)	241(25.8)
	Year 3	90(21.9)	218(23.3)
	Year 4	56(13.6)	157(16.8)
	Year 5	11(2.7)	20(2.1)
	Year 6	12(2.9)	22(2.4)
**Age**	Mean = 21.87	Standart Deviation = 2.91	

### Data analysis

#### Stage 1

It was explored whether or not there was any missing value in the data obtained from the application. A number of missing values were found in the data file. Thus, it was investigated whether the missing values were totally random or systematic. The randomness of missing data was studied on SPSS with “Estimate Mean (EM)”. The missing data were found to be random (*p* > 0.05). The missing data that were found to be random were then completed by a mean rank of the respective variable. After the completion of the missing value replacement, multivariate extreme values ​​in the data file were determined. For this, the Mahalanobis distance was calculated since this method is a popular multivariate distance metric which investigates the actual distance between a single point and the distribution. The detected extreme values ​​were removed from the data file. Next, if the data file was suitable for a factor analysis was tested with Kaiser Meyer Olkin (KMO) Test and Bartlett’s Test of Sphericity. Possible factorizations that may come up in the factor analysis were tested with “Oblimin Rotation of the Axis”. The CFA evidence obtained in the study were examined with goodness of fit indices. According to CTT, validity of the scale was examined in the context of construct validity (in line with factorization and eigenvalue values ​​depicted by the literature). Also, according to CTT, reliability of the scale could be examined in the context of internal consistency.

#### Stage 2

The data of the medical school students obtained from DESS were analysed in terms of validity and reliability on the basis of IRT. Moreover, within the scope of IRT, validity of the scale was examined considering the item discrimination and item difficulty levels. Additionaly, the reliability within the scope of IRT was examined with the Marginal Confidence coefficient. The unidimensionality and local independence assumptions were needed to be investigated when testing validity and reliability with IRT for the items requiring a rated response set (for instance Likert Type). Unidimensionality was examined using EFA in this study. Local independence assumption was tested using the Q3 statistic [31]. The IRT calibrations were established using the “mirt v. 1.30” [32] package on the R v. 3.5.0 software.

#### Stage 3

**Fig. 1:**
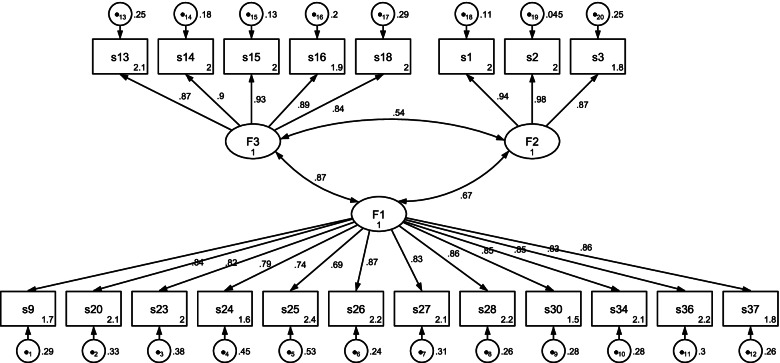
CFA results of DESS

Measurement invariance was realised through the “lavaan” package [33]. Measurement invariance was realised for males and females according to gender. Gender variable here in this study is critical since the role of gender is very important in the satisfaction of medical students with distance education in the context of Turkey. It is a fact that in some regions, female students cannot move freely compared to males. Moreover, they have to do housework and/or take care for younger siblings at home etc. Since this study was conducted in Turkish context, the gender variable was particularly emphasized because female students could be under more pressure and may find less time to study (for the aforementioned house chores) compared to male students who have much more space for studying and individual freedom. The results were tested separately for female and male students through CFA using the measurement model presented in Fig. 1. As CFA goodness of fit indices, the chi-square/ degree of freedom, RMSEA, CFI and TLI were used. Kline [25] has reported that the chi-square/ degree of freedom should be 3 or a value less than that. Tabachnick and Fidell [34] state that this value should be 0.080 or lower in CFA as a RMSEA goodness of fit indicator. The CFI and TLI values being larger than 0.95 indicates that CFA goodness of fit has been achieved [35]. After CFA, “configural invariance (equal form)”; “metric invariance (equal factor loadings)”, “scalar invariance (equal indicator intercepts)” and “strict factorial invariance (equal indicator error variances)” were tested for invariance for female and male students. In testing of measurement invariance, Cheung and Rensvold [36] and Chen [37] state that the criterion for delta CFI should be equal to or less than 0.01. The criterion was taken as ΔCFI ≤ 0.01 in this study.

## Results

### Stage 1

#### DESS factor construct

At the first stage of the analysis, the construct of the 37-item and 7-factor DESS developed by Karadağ et al. was modelled by the “mirt” package using the multidimensional modelling on the R software. However, since analysis could not be finalized despite reaching 500 iterations, the multidimensional IRT analysis was abolished.

Karadağ et al. [18] developed DESS based on the data obtained from university students studying in different departments and obtained validity and reliability evidences. This study, however, used the data obtained from only medical school students studying in different universities. Assuming that “If the scale could revel a different factor construct?”, another factor analysis process was carried out. As a result of the new EFA performed with the data of the medical faculty students, the KMO value was calculated as 0.959 and the Bartlett's Test of Sphericity value was found as 8733.83 (*p* < 0.05). The factor analysis was performed using the principal axis factoring-PAF [38], and the rotation using the oblimin method, one of the oblique rotation methods. The number of factors was determined considering the eigenvalue values ​​predicted by the literature. Items 4, 5, 6, 7, 8, 10, 11, 12, 17, 19, 21, 22, 29, 31, 32, 33 and 35 that exhibited a low total item correlation and a high correlation under more than one factor were removed and a construct consisting of 3 factors and 20 items was obtained. This 3-factor construct explained 73.5% of the total variance for DESS. This is quite a high variance explanatory level according to the literature [34, 39, 40]. In the items grouped under the factors in the 3-factor construct obtained, the factor loads did not decrease below 0.692 and the total item correlations were not below 0.636 (See Attachment 1). The Cronbach Alpha reliability levels of these 3 factors were 0.96, 0.95 and 0.95, and that of the whole scale was 0.97. This 3-factor construct was validated by the fit indices in the CFA analysis (RMSEA = 0.078, CFI = 0.952, TLI = 0.945). The finalized diagram is shown in Fig. 1.

The newly obtained 3-factor and 20-item construct of DESS was tested by the “mirt” package using the multidimensional modelling on the R software. Assuming each of the 3 factors were locally independent, they were modelled individually using the mirt package on the R software.

### Stage 2

#### Validity and reliability evidence of DESS based on IRT

The data of the medical school students obtained from DESS were studied for validity and reliability evidence based on IRT. To be able to use IRT, certain assumptions should be studied. The first of these assumptions, unidimensionality, was explored with EFA. In educational psychology, measurement constructions (satisfaction in this research) are mostly assumed to be theoretically multidimensional. Results of the factor analysis confirms this hypothesis. In factor analysis and scale development, researchers should test different models of the measurement tool, including single-factor and multi-factor models. For example, if the factor correlations indicate that the scale is suitable for the holistic structure (high correlation between sub-dimensions), a single factor model can also be accepted [41]. EFA revealed that the sub-factors in the 20-item 3-factor construct of DESS were highly correlated with each other. But the analysis was performed assuming that each sub-factor was independent. Local independence was determined using the Q3 statistic as suggested by Yen [31]. According to the Q3 statistic, there were no items impairing local independence among the 12 items included in the first factor of the scale, the 3 items included in the second factor, or the 5 items included in the third factor. Item calibrations were determined with IRT, generalized partial credit model (GPCM) for the items in all three sub-factors.

Commonly used IRT models developed for items with two or more sequential categories (for example, Likert-type scale structures) could be listed as Partial Credit Model (PCM) and Generalized Partial Credit Model (GPCM). The reason why GPCM is preferred over PCM is that it predicts the item discrimination parameter in addition to the item difficulty parameter [42, 43]. The S_χ2, degree of freedom, RMSEA, and level of significance statistics of the items were calculated in line with the GPCM. The results are shown in Table 2.

**Table 2:** Item fit indexes as per IRT

Factor			GPCM		
**F1**	**F2**	**F3**	**S_χ**^**2**^	**df**	**RMSEA**
s9			370.5	328	0,012
s20			429.9	324	0,019
s23			393.9	327	0,015
s24			432.8	311	0,020
s25			368.9	294	0,017
s26			337.2	267	0,017
s27			412.9	281	0,022
s28			346.2	285	0,015
s30			359.9	267	0,019
s34			328.2	283	0,013
s36			396.0	281	0,021
s37			390.2	290	0,019
	s1		40.0	25	0,025
	s2		85.5	24	0,052
	s3		80.0	35	0,037
		s13	180.4	131	0,020
		s14	147.9	117	0,017
		s15	139.9	97	0,022
		s16	190.2	124	0,024
		s18	208.0	149	0,021

The most important goodness of fit statistic in IRT is RMSEA. The limit value for RMSEA is 0.08 and a value less than this indicates goodness of fit [19, 20]. According to the goodness of fit statistics in Table 2, the RMSEA values of the items are less than 0.08. Based on this result, it was concluded that the scale construct obtained with EFA satisfied model fit as per GPCM. The “a” (item discrimination) and “b” (item difficulty) parameters and standard errors of the items matched with the model fit as per GPCM were estimated. The results are shown in Table 3.

**Table 3:** Item parameters and standard error values as per GPCM

**Factor**			**a(SE)**	**b1(SE)**	**b2(SE)**	**b3(SE)**	**b4(SE)**	**b5(SE)**	**b6(SE)**	**b7(SE)**	**b8(SE)**	**b9(SE)**
**F1**	**F2**	**F3**										
s9			1.12(0.08)	-0.27(0.14)	-0.69(0.15)	-0.35(0.15)	-0.47(0.14)	0.19(0.15)	-1.14(0.15)	0.28(0.14)	0.72(0.16)	0.39(0.15)
s20			1.09(0.07)	-0.64(0.17)	-0.83(0.17)	-0.70(0.16)	-0.58(0.15)	-0.17(0.15)	-0.19(0.15)	-0.04(0.14)	0.42(0.14)	0.30(0.13)
s23			0.84(0.06)	0.31(0.24)	-0.98(0.25)	-0.83(0.22)	-0.95(0.19)	0.10(0.18)	-0.07(0.19)	-0.02(0.18)	0.61(0.19)	-0.12(0.18)
s24			0.59(0.04)	1.52(0.30)	-0.14(0.34)	-0.10(0.37)	-1.95(0.34)	0.71(0.27)	-0.13(0.29)	0.11(0.28)	0.81(0.28)	-0.46(0.27)
s25			0.61(0.05)	0.78(0.39)	-1.24(0.42)	-0.73(0.40)	-1.64(0.35)	-0.54(0.28)	-0.06(0.28)	-0.44(0.27)	-0.33(0.25)	-1.51(0.23)
s26			1.62(0.11)	-0.68(0.13)	-0.70(0.14)	-0.90(0.14)	-0.86(0.11)	0.08(0.11)	-0.43(0.11)	-0.12(0.09)	0.41(0.09)	0.32(0.09)
s27			1.22(0.08)	0.09(0.19)	-1.12(0.20)	-0.67(0.17)	-1.08(0.15)	-0.02(0.12)	-0.29(0.13)	0.09(0.12)	0.59(0.13)	0.13(0.13)
s28			1.57(0.11)	-0.68(0.13)	-0.71(0.14)	-0.83(0.14)	-0.70(0.12)	-0.36(0.10)	-0.17(0.10)	-0.21(0.10)	0.33(0.10)	0.23(0.10)
s30			1.31(0.09)	0.13(0.12)	-0.41(0.13)	0.02(0.15)	-0.69(0.15)	0.19(0.13)	-0.12(0.13)	0.46(0.13)	0.86(0.15)	0.32(0.15)
s34			1.36(0.09)	-0.43(0.15)	-1.09(0.16)	-0.36(0.15)	-1.07(0.14)	0.05(0.12)	-0.44(0.12)	-0.12(0.11)	0.56(0.11)	0.27(0.11)
s36			1.24(0.08)	-0.43(0.17)	-0.77(0.18)	-0.52(0.19)	-1.46(0.17)	-0.03(0.12)	-0.15(0.13)	-0.29(0.13)	0.23(0.12)	0.16(0.11)
s37			1.34(0.09)	-0.10(0.13)	-0.68(0.15)	-0.39(0.14)	-0.79(0.13)	0.18(0.13)	-0.26(0.13)	0.18(0.12)	0.59(0.13)	0.30(0.13)
	s1		3.39(0.32)	-1.01(0.14)	-0.85(0.15)	-0.57(0.14)	-0.50(0.10)	0.13(0.13)	0.22(0.13)	0.41(0.10)	0.94(0.13)	0.99(0.13)
	s2		5.43(0.15)	-1.16(0.11)	-0.88(0.15)	-0.63(0.14)	-0.37(0.09)	0.02(0.13)	0.17(0.13)	0.54(0.09)	0.86(0.13)	1.14(0.15)
	s3		1.33(0.17)	-0.44(0.12)	-0.77(0.15)	-0.50(0.14)	-0.42(0.11)	0.29(0.13)	0.10(0.13)	0.83(0.13)	1.07(0.13)	0.74(0.13)
		s13	1.31(0.09)	-0.72(0.15)	-1.13(0.15)	-0.52(0.14)	-0.60(0.14)	-0.43(0.12)	-0.01(0.11)	0.15(0.11)	0.52(0.12)	0.57(0.12)
		s14	2.01(0.15)	-0.73(0.10)	-0.73(0.11)	-0.48(0.11)	-0.65(0.10)	-0.36(0.09)	-0.06(0.08)	-0.03(0.08)	0.51(0.08)	0.66(0.08)
		s15	2.87(0.24)	-0.92(0.08)	-0.82(0.08)	-0.62(0.08)	-0.45(0.07)	-0.27(0.07)	-0.17(0.06)	0.15(0.06)	0.55(0.07)	0.76(0.07)
		s16	1.77(0.13)	-0.85(0.10)	-0.65(0.11)	-0.52(0.11)	-0.50(0.11)	-0.12(0.10)	-0.26(0.10)	0.17(0.10)	0.47(0.10)	0.48(0.10)
		s18	1.08(0.08)	-0.28(0.17)	-0.73(0.19)	-0.63(0.18)	-0.63(0.17)	-0.18(0.16)	-0.32(0.15)	-0.01(0.14)	0.54(0.14)	0.22(0.14)
Iteration=339LogLikelihood= -21265.183*p*<.05					Iteration=234LogLikelihood= -4783.598*p*<.05				Iteration=111LogLikelihood= -10174.704*p*<.05			

**Fig. 2:**
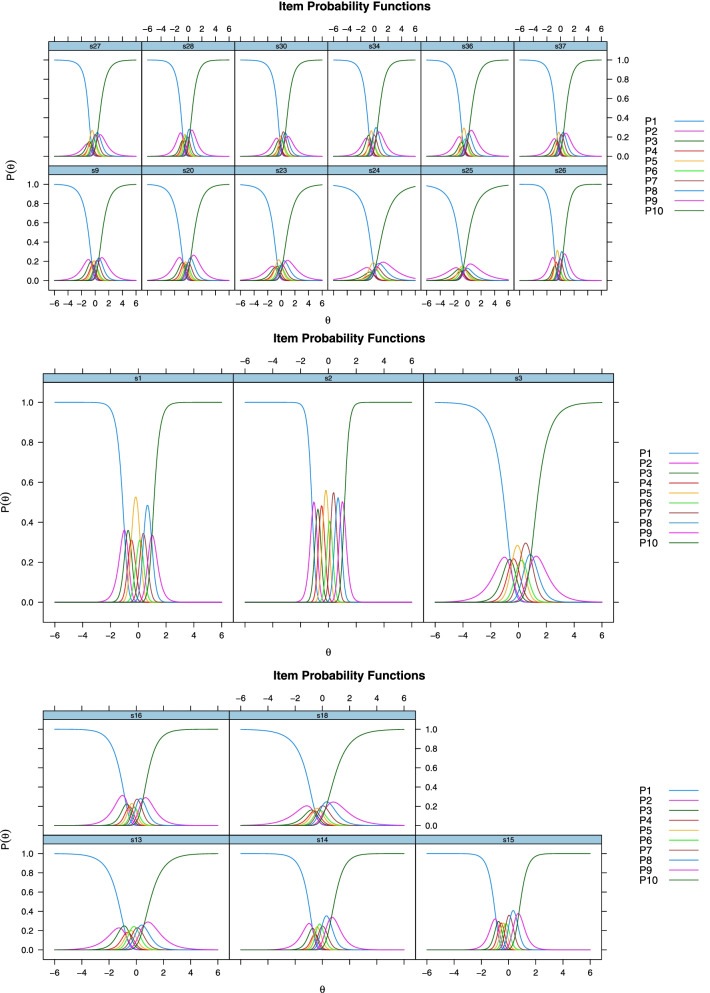
Characteristic curves for DESS items

The estimations made according to GPCM (LogLikelihood, *p* < 0.05) seem to evidence the fit of scale items [32]. The item characteristic curves are shown in Fig. 2. In IRT, the distinctiveness value of an ideal scale item (ie the "a" parameter) should be between 0.5 and 2. In the literature, it is accepted that the required parameter is between 0.75 and 2.50 [44]. Findings in Table 3 ​​show that the discrimination values ​​of items s3, s9, s13, s14, s16, s18, s20, s23, s26, s27, s28, s30, s34, s36 and s37s are at the desired level. The ideal (medium difficulty level) limits for item difficulty levels (ie, the "b" parameter) are considered to be between -1.00 and 1.00 [45].

**Fig. 3:**
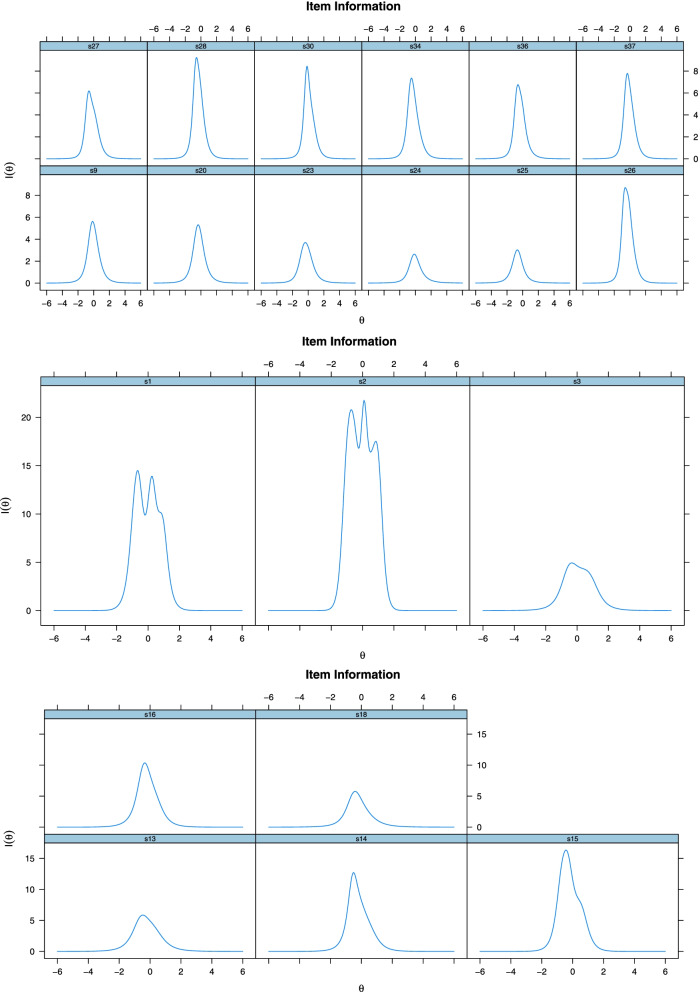
Information Functions of DESS Items

The item characteristic curves in Fig. 2 show that the items included in the scale together with their options had a good performance for different levels of ability and proved to be discriminative. The discriminations of the response categories of Items 24 and 25 was relatively lower compared to the other items. The response categories of the items in the scale were understood by the participants and had a discriminative function. The item information function is a graphical representation showing the range of the feature (the feature tried to be measured on the scale namely “*student satisfaction*” in this study) that best distinguishes the individuals who participated in the study [46]. In the item information function, the higher the peak of the curve, the more information the item is interpreted. Item information functions are shown in Fig. 3.

**Fig. 4:**
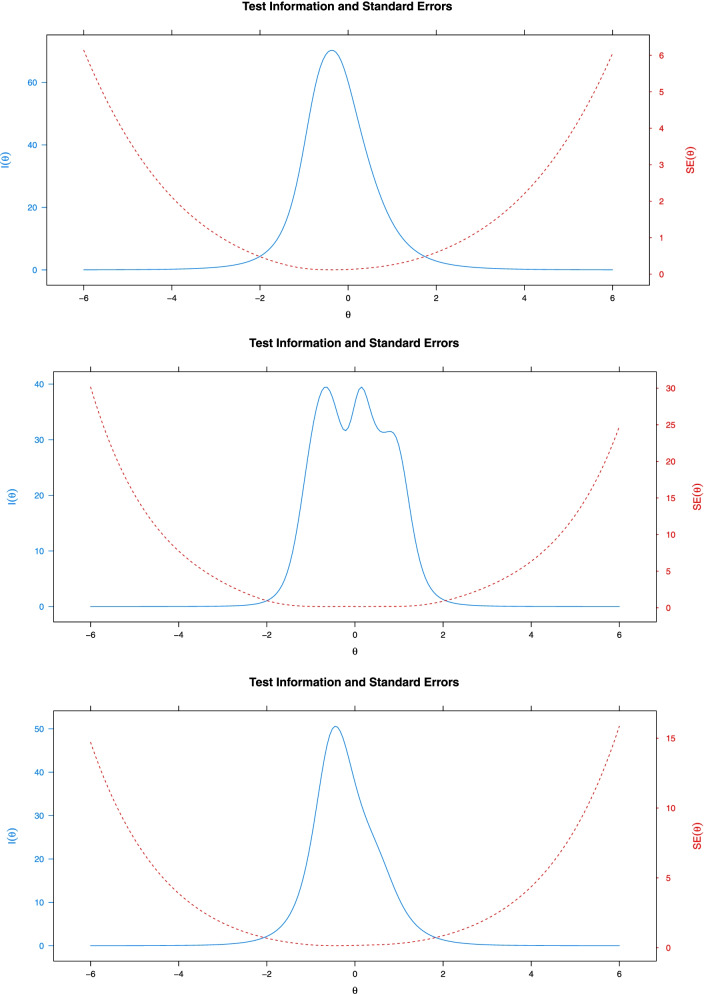
Information Functions of DESS Sub-Factors

A review of the item information functions of DESS items revealed that the least informative items were item 23, 24 and 25 in Factor 1, item 3 in Factor 2, and items 13 and 18 in Factor 3. The test information function is shown in Fig. 4.

The test information function shows the level of giving information about the feature of the measurement tool as a whole [17]. The level at which all 3 factors of the scale give best information about the items is the -2 and 2 intervals. As in Fig. 4, its appearance similar to the normal distribution curve is an indication that it provides information for different levels of the measured feature, since a scale provides the best information for individuals’ satisfaction levels in this interval. The marginal reliability coefficient of the scale was calculated to be 0.94 for F1, 0.92 for F2 and 0.91 for F3. These values are quite close to the reliability values obtained with Cronbach Alpha.

### Stage 3

#### Measurement invariance evidence of dess with respect to genders of medical school students

The measurement model in Fig. 1 was tested for female and male students using CFA. The CFA fit indices obtained separately for male and female students are given in Table 4.

**Table 4:** CFA fit indices for male and female students

Gender	X^2^/ df	RMSEA	CFI	TLI
Female	2.73	0,073	0,957	0,953
Male	2.94	0,077	0,950	0,948

The 3-factor construct with a measurement model shown for both male and female students in Fig. 1 works within the framework of the CFA indices that are acceptable in the literature and using the same construct [19–24]. After finding that it works for both female and male students in a similar construct, DESS was tested for invariability among the students of both genders. Each dimension of measurement variance; configural, metric, scalar and strict invariance were tested. The fit indices and ΔCFI values obtained from the results were reviewed. The results were summarized in Table 5.

**Table 5:** Measurement invariance data for male and female students

Measurement invariance	RMSEA	CFI	TLI	ΔCFI
Configural	0,080	0,949	0,942	
Metric	0,079	0,949	0,945	0,000
Scalar	0,077	0,948	0,947	0,001
Strict	0,076	0,942	0,941	0,006

DESS works in the same factor construct for both genders. The results obtained for metric, scalar and strict invariance showed that the ΔCFI value was smaller than 0.01. This showed that there was invariance between female and male students in terms of DESS factor construct and factor loads [36, 37].

## Discussion

The factor construct of DESS, which had been studied for all higher education students before, was studied particularly for medical school students in this study. Instead of the original construct of the scale consisting of 37 items and 7 sub-factors, a construct with 20 items and 3 sub-factors, which produced good statistical results for medical school students was discovered. A multidimensional review of this construct on the basis of IRT resulted in too many iterations. Considering this result, it was made subject to a unidimensional IRT analysis assuming that each factor of the 20-item, 3-sub-factor construct was locally independent. According to the IRT analysis, the most informative items were items 9, 20, 26, 27, 28, 30, 34, 36 and 37 in Factor 1 and items 1 and 2 in Factor 2, and the least informative items were 14, 15 and 16 in Factor 3. These items were;

- Item 1: With the decisions made by CHE…
- Item 2: With the attitudes and approaches of CHE…
- Item 9: With the teaching capacity of digital content/teaching materials…
- Item 14: With the attitudes of the lecturers towards students…
- Item 15: With the teaching skills of the lecturers…
- Item 16: With the information provided by the lecturers on the process…
- Item 20: With the accessibility of the distance education system…
- Item 26: With the student–lecturer communication in synchronized/live courses…
- Item 27: With the duration of lessons in synchronized courses…
- Item 28: With the accessibility of distance education course contents/materials…
- Item 30: With the efficiency of distance education courses…
- Item 34: With the conduct of courses in line with predetermined plans…
- Item 36: With the extent to which opportunity for student questions and participation is given during classes…
- Item 37: With the method of assessing my achievement and the sufficiency of such method…

The study of Sahin and Shelley [47] has shown that students’ computer knowledge and perceived flexibility and practicability of distance education were the predictors of their achievement in an online learning environment. They mentioned about “flexibility and practicability” as the most informative items of DESS in their study. Weidlich and Bastiaens [48] and Giannousi et al. [49] have demonstrated that having meaningful interest in the content of learning and feeling a certain psychological closeness towards the educator are necessary for a satisfactory experience. They mentioned about the ability to interact with the content and the role of lecturers as the highly informative items of DESS in their study.

At the second stage of the study, the measurement invariability of the scale was tested. The fit indices obtained in the testing of DESS construct for “configural” invariability were acceptable. Fulfilment of configural invariability shows that female and male medical school students perceive satisfaction from distance education in a similar way. Cole et al. [50] found that university students’ satisfaction from online courses did not differ significantly with respect to gender. Sapanca [51] concluded that there was no statistically significant difference between the attitudes and interests of university students towards online learning with respect to gender. Some other studies [52–54] have also found similar results. Liaw and Huang [55], on the other hand, found that male students had a more positive attitude towards online learning than female students.

Metric invariability was also fulfilled between female and male medical school students. Metric invariability is obtained when different groups respond similarly to the items in the scale. This means that the relationship between the items and the construct of the scale has similar significance between the groups. Once metric invariability is achieved, the scores obtained from the items can be compared between the groups and the variation in the items may show the differentiation between the groups in terms of measured construct [56]. Metric invariability is a prerequisite for testing the scalar invariability analysis. Scalar invariability should be obtained to compare mean scores [56]. Scalar invariability is a must for the comparison of latent constructs (latent variables in factor analysis) between the groups. The scalar invariability of the DESS construct was fulfilled for both genders. Strict invariance means that the conditional variance of the responses to common factors received from the participants is equivalent among the female and male medical school students. Strict invariance requires that the factor loads, item regression constants and residual variances are equivalent between the groups [57]. Strict invariability was also fulfilled in the study. The results revealed that it is possible to make comparisons in terms of gender as it was also conducted by DESS.

### Limitations

The major limitation of this study was that the 37-item and 7-sub-factor construct, and the 20-item and 3-sub-factor construct could not be analyzed at the iteration limit required by the multidimensional IRT analysis. For this reason, each sub-factor of the scale was assumed to be locally independent, and they were processed as if they were single-factor scales. Experimenting the multidimensional IRT modelling on data to be obtained from different groups in future studies will provide a great contribution to the development of the scale.

## Supplementary Information

**Additional file 1.** DESS EFA Data: The EFA includes applied data.

**Additional file 2.** DESS CFA Data: The CFA includes applied data.

**Additional file 3.** F1 IRT Data: It includes data for which IRT is applied to factor 1.

**Additional file 4.** F2 IRT Data: It includes data for which IRT is applied to factor 2.

**Additional file 5.** F3 IRT Data: It includes data for which IRT is applied to factor 3.

**Additional file 6.** Attachment 1: Factor loading and corrected item correlation values of items under three factors as a result of EFA.

## Data Availability

The datasets used analyzed during the study are available from the corresponding author on reasonable request. The datasets generated during and analyzed during the current study are not publicly available due to (The research data sets generated and analyzed during the current study are not publicly available due to the confidentiality announcement made on the participants but are available from the corresponding author on reasonable and ethical request.) but are available from the corresponding author on reasonable request. We, as authors, hereby confirm that all methods were performed in accordance with the relevant guidelines and regulations stated in Declaration of Helsinki.
