# Supplementary material for: Validity and reliability evidence for the scale of distance education satisfaction of medical students based on item response theory (IRT)
Source: BMC Med Educ. 2022 Feb 11;22:94. doi: 10.1186/s12909-022-03153-9 (PMC8831879; doi:10.1186/s12909-022-03153-9)
Supplement: Supplementary file 6 — Additional file 6. Attachment 1: Factor loading and corrected item correlation values of items under three factors as a result of EFA. [file 12909_2022_3153_MOESM6_ESM.docx]

**Appendix 1**

|  | **Factor** | | | | | |
| --- | --- | --- | --- | --- | --- | --- |
|  | **F1: Satisfaction with Distance Education** | | **F2: Satisfaction with YÖK's Approach in Distance Education** | | **F3: Satisfaction with Instructors in Distance Education** | |
|  | **Direct Oblimin Rotation Correlation** | **Corrected Item-Total Correlation** | **Direct Oblimin Rotation Correlation** | **Corrected Item-Total Correlation** | **Direct Oblimin Rotation Correlation** | **Corrected Item-Total Correlation** |
| **s28:** With the accessibility of distance education course contents/materials... | 0.897 | 0.846 |  |  |  |  |
| **s26:** With the student-lecturer communication in synchronized/live courses... | 0.868 | 0.832 |  |  |  |  |
| **s30:** With the efficiency of distance education courses... | 0.862 | 0.834 |  |  |  |  |
| **s27:** With the duration of lessons in synchronized courses... | 0.850 | 0.803 |  |  |  |  |
| **s34:** With the conduct of courses in line with predetermined plans... | 0.835 | 0.816 |  |  |  |  |
| **s9:** With the teaching capacity of digital content/teaching materials... | 0.833 | 0.841 |  |  |  |  |
| **s37:** With the method of assessing my achievement and the sufficiency of such method... | 0.823 | 0.824 |  |  |  |  |
| **s20:** With the accessibility of the distance education system... | 0.822 | 0.810 |  |  |  |  |
| **s36:** With the extent to which opportunity for student questions and participation is given during classes... | 0.817 | 0.808 |  |  |  |  |
| **s23:** With the process of uploading homework to the system... | 0.778 | 0.773 |  |  |  |  |
| **s24:** With online examination procedures... | 0.699 | 0.688 |  |  |  |  |
| **s25:** With accessibility to course recordings for repetition purposes... | 0.692 | 0.639 |  |  |  |  |
| **s2:** With the attitudes and approaches of CHE... |  |  | 0.980 | 0.657 |  |  |
| **s1:** With the decisions made by CHE... |  |  | 0.920 | 0.687 |  |  |
| **s3:** With the explanations and instructions of CHE... |  |  | 0.885 | 0.636 |  |  |
| **s15:** With the teaching skills of the lecturers... |  |  |  |  | 0.922 | 0.818 |
| **s16:** With the information provided by the lecturers on the process... |  |  |  |  | 0.902 | 0.807 |
| **s14:** With the attitudes of the lecturers towards students... |  |  |  |  | 0.897 | 0.783 |
| **s13:** With the technology utilization skills of the lecturers... |  |  |  |  | 0.848 | 0.788 |
| **s18:** With the ease of contacting lecturers... |  |  |  |  | 0.848 | 0.775 |
